# A redox-responsive dihydroartemisinin dimeric nanoprodrug for enhanced antitumor activity

**DOI:** 10.1186/s12951-021-01200-z

**Published:** 2021-12-20

**Authors:** Yawei Li, Qing Pei, Baiji Cui, Hongmei Zhang, Liu Han, Wenqing Li, Wenhe Zhu, Xianmin Feng, Zhigang Xie

**Affiliations:** 1 Jilin Medical University, Jilin, 132013 People’s Republic of China; 2 State Key Laboratory of Polymer Physics and Chemistry, Changchun Institute of Applied Chemistry, Chinese Academy of Sciences, Changchun, 130022 People’s Republic of China

**Keywords:** Dimeric nanoprodrug, Dihydroartemisinin, Redox-responsive, Antitumor activity, PI3K/AKT/HIF-1α signaling pathway

## Abstract

Redox-responsive drug delivery system emerges as a hopeful platform for tumor treatment. Dihydroartemisinin (DHA) has been investigated as an innovative tumor therapeutic agent. Herein, a DHA dimeric prodrug bridged with disulfide bond as linker (DHA_2_-SS) has been designed and synthesized. The prepared prodrugs could self-assemble into nanoparticles (SS NPs) with high DHA content (> 90%) and robust stability. These SS NPs display sensitive redox responsive capability and can release DHA under the tumor heterogeneity microenvironment. SS NPs possess preferable antitumor therapeutic activity in contrast with free DHA. Moreover, the possible anti-cancer mechanism of SS NPs was investigated through RNA-seq analysis, bioinformatics and molecular biological method. SS NPs could induce apoptosis via mitochondrial apoptosis pathway, as well as glycolysis inhibition associate with the regulation of PI3K/AKT/HIF-1α signal path, which may offer an underlying therapeutic target for liver cancer. Our study highlights the potential of using redox responsive prodrug nanoparticles to treat cancer, meanwhile provides insights into the anti-cancer mechanism of DHA prodrug.

**Supplementary Information:**

The online version contains supplementary material available at 10.1186/s12951-021-01200-z.

## Introduction

Currently, chemotherapy remains the predominant therapy in cancer treatment due to its versatility and high efficiency [1–4]. For the past few years, traditional herbal medicines have generated many chemotherapy drugs which could inhibit a variety of tumor entities [5]. For instance, dihydroartemisinin (DHA), one derivative of artemisinin, has been proved to possess a potent and broad anti-tumor effect in addition to anti-malarial [6–9]. However, some existing problems, for example strong hydrophobicity, nonspecific distribution, and rapid elimination from the body, impede the application of DHA in cancer treatment [10–12]. To overcome these limitations, nano drug-delivery systems have been employed to increase the solubility, prolong systemic circulation, and promote passive tumor targeting owing to EPR effect [13–17]. However, low drug content and uncontrolled drug release of these nanoparticle formulations are still far from satisfactory [18–20]. Hence, developing effective nanoplatforms with the tumor microenvironment-responsive drug release and high drug content is highly desirable for various chemotherapeutic drugs.

In recent years, growing evidences demonstrate that dimerization of drug molecules has emerged as a powerful tool for developing new prodrugs [16, 21–25], and organic dimers are liable to self-assemble into NPs in aqueous solution [18, 25–30]. In addition, the drug-delivery system should also possess the responsive and controllable drug release, which can realize specific selectivity towards tumor cells and the low side effects [31–36]. As we known, in contrast with normal cells, tumor cells usually exhibit higher levels of intracellular glutathione (GSH) and reactive oxygen species (ROS) [37–40]. A variety of redox-responsive chemical bonds have been developed to fabricate the tumor microenvironment-responsive nanomaterials [41–46]. Therefore, it is of great significance to design DHA nanoprodrug with redox-responsive linker, which can effectively resolve the existing problems of DHA and further improve its therapeutic effects.

**Scheme 1:**
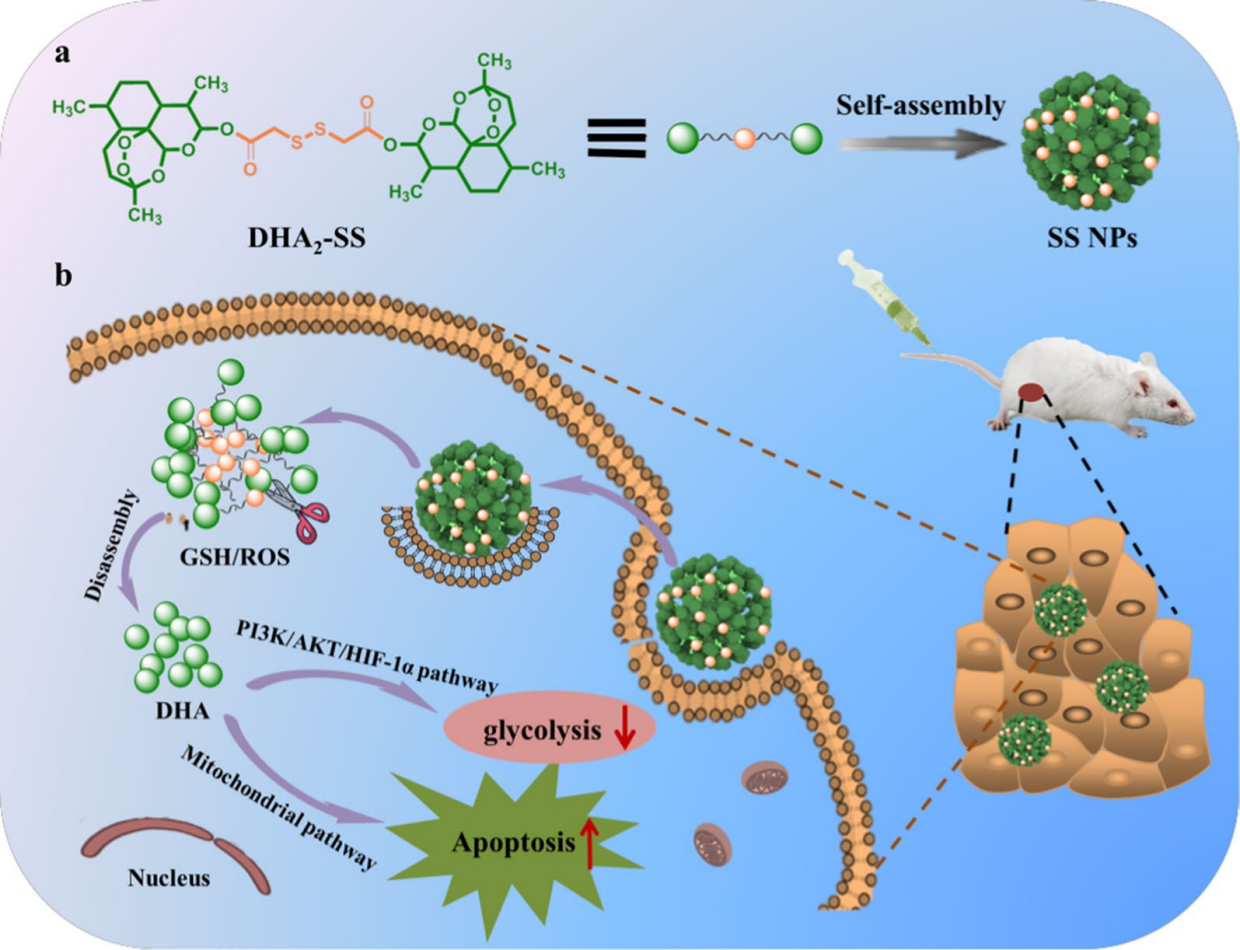
Schematic illustration of (**a**) the construction of disulfide bond linker-bridged dihydroartemisinin dimeric prodrug nanoparticles (SS NPs) and (**b**) their application for antitumor therapy

In our present work, a DHA dimer containing disulfide bond linker, which has been proven to possess dual-responsiveness [47–49], was designed and successfully synthesized, named as DHA_2_-SS. And this DHA dimer could self-assembly into nanoparticles (SS NPs) in aqueous media by nanoprecipitation method. The formed SS NPs possess nanoscale size, robust stability, and ultra-high drug content. The redox response, cellular uptake and antitumor efficacy of SS NPs have been studied, and the gene expression of tumor cells after SS NPs treatment was analyzed by RNA-seq analysis (Scheme 1).

## Results and discussion

### Preparation and characterization of DHA dimeric NPs

Firstly, the DHA_2_-SS was synthesized through the esterification reaction of DHA with dicarboxylic acid (Additional file 1: Fig. S1) [23]. After purification by silica gel column chromatography, DHA_2_-SS was obtained in high yields (> 90%) and the chemical construction has been characterized via proton nuclear magnetic resonance (^1^H NMR) spectroscopy and a linear ion trap mass spectrometer (LTQ-MS). In ^1^H NMR spectra, the disappearance of proton of 10-hydroxyl group from DHA at around 4.7 ppm and the appearance of new peaks near 3.66 ppm validated the success of esterification reaction, and the reaction site was at the 10-hydroxyl group of DHA (Additional file 1: Fig. S2). The peak value corresponding to DHA_2_-SS at around 737 in mass spectrometry was consistent with the theoretical calculated value (Additional file 1: Fig. S3), further confirming the structure of DHA dimer. In order to compare the redox responsiveness of DHA_2_-SS, we also synthesized another DHA dimer with the same length of carbon chain (DHA_2_-C6) as control (Additional file 1: Figs. S1, S2 and S4).

**Fig. 1:**
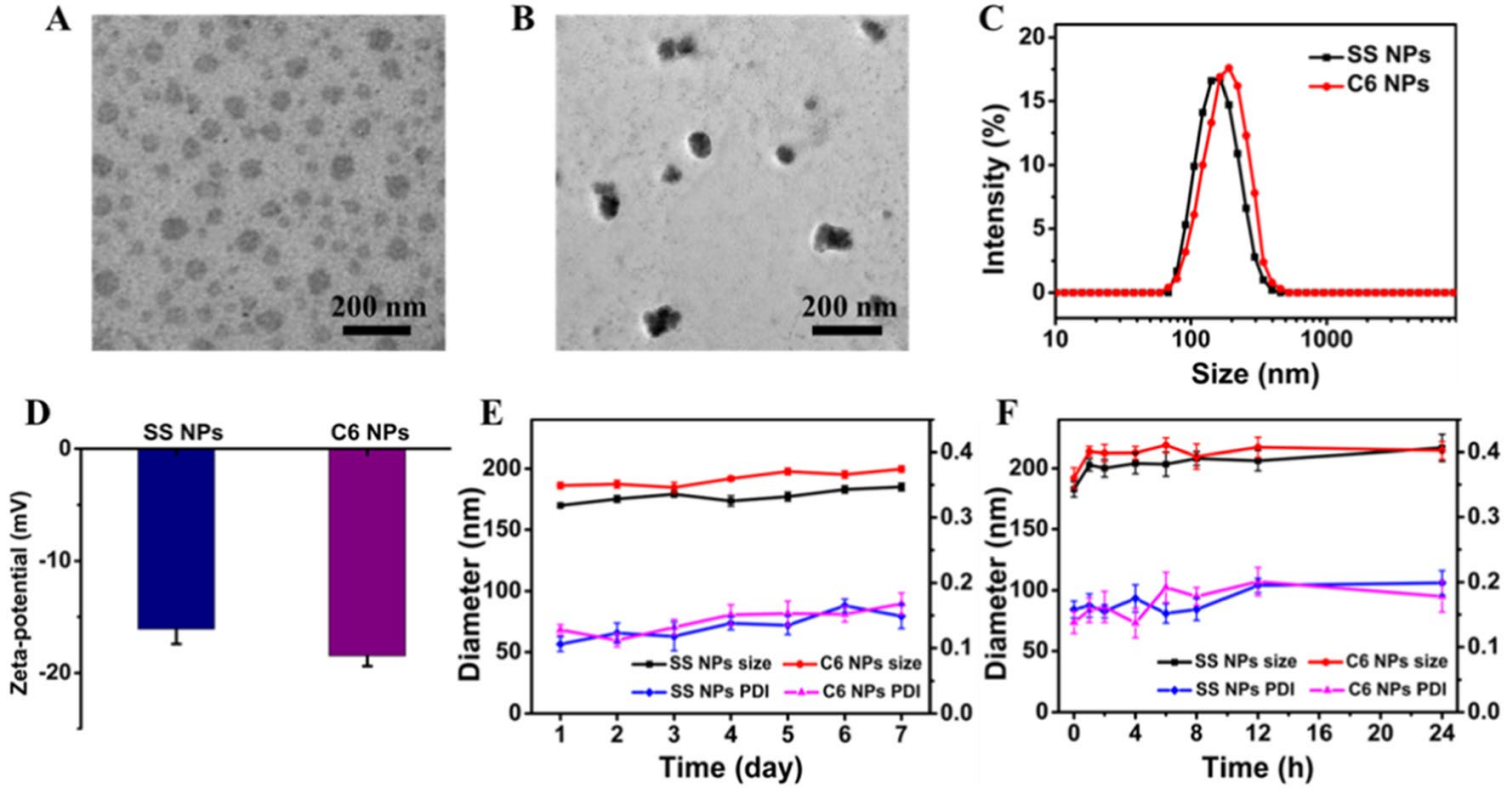
Basic characterization of DHA dimeric NPs. TEM images of (**A**) SS NPs and (**B**) C6 NPs. **C** Size distribution and (**D**) zeta potential of prepared SS and C6 NPs. Changes of hydrodynamic diameter and PDI of two kinds of NPs (**E**) in water and (**F**) in PBS with FBS (10%) over different times measured by DLS. Data are expressed as mean ± SD (n = 3)

It is reported that the organic dimers could self-assemble into NPs in aqueous solution [25–30]. As anticipated, both kinds of resulting dimers formed spherical nanoparticles (abbreviated as SS NPs and C6 NPs, respectively) through nanoprecipitation method as observed via transmission electron microscopy (TEM) (Fig. 1A, B). And SS NPs had an average hydrodynamic diameter of approximately 167.2 nm as determined by dynamic light scattering (DLS), which was similar to those of C6 NPs (181.4 nm) (Fig. 1C). These two kinds of NPs were found to be negative, and the zeta potential values were around − 20 mV (Fig. 1D). The drug content of SS and C6 NPs was 90.6% and 91.7%, respectively. In addition, SS and C6 NPs both possessed robust stability with negligible changes in size and size distribution in one week (Fig. 1E), and also kept stable in PBS (pH 7.4) containing 10% FBS after 24 h (Fig. 1F), in which a slight size increase in the first two hours was ascribed to the protein absorption on the surface of NPs. Furthermore, these two kinds of NPs also had good structural stability after different treatments (Additional file 1: Figs. S5, S6 and S7).

### DTT and GSH triggered release of DHA

**Fig. 2:**
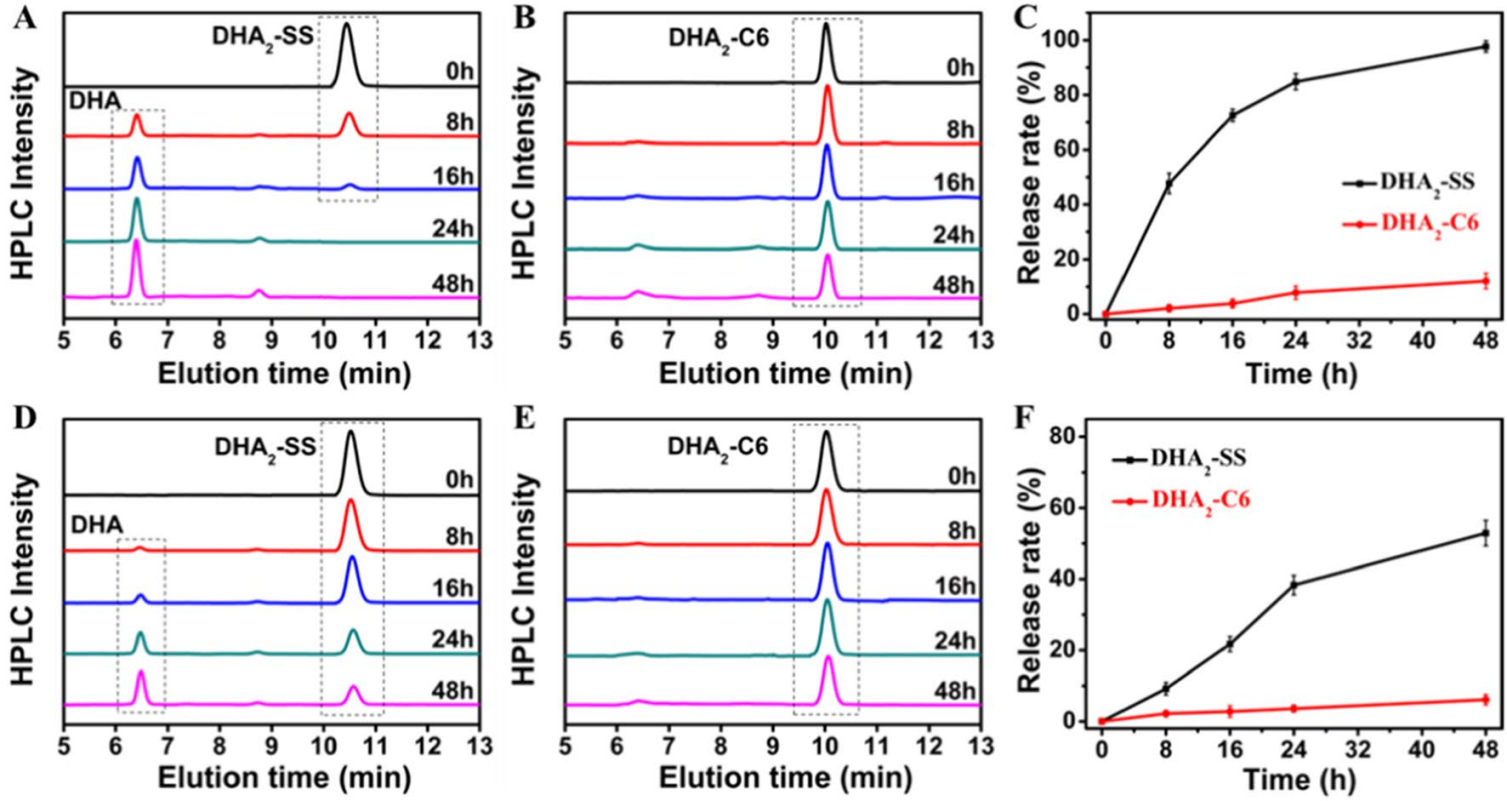
Reduction responsiveness of (**A**) DHA_2_-SS and (**B**) DHA_2_-C6 dimers degradation and (**C**) the rate of DHA released from DHA dimers in the presence of 10 mM DTT at 37 °C. Oxidation responsiveness of (**D**) DHA_2_-SS and (**E**) DHA_2_-C6 dimers degradation and (**F**) the rate of DHA released from DHA dimers in the presence of 10 mM H_2_O_2_ at 37 °C

As mentioned above, disulfide bond possess distinct redox response capability [47–49]. Therefore, we investigated the responsiveness of DHA_2_-C6 and DHA_2_-SS by using dithiothreitol (DTT) and H_2_O_2_ as reducing and oxidizing agents, respectively. As shown in Fig. 2A, after incubation with 10 mM DTT, the HPLC peak of DHA_2_-SS at 10.6 min declined significantly and entirely disappeared in 24 h, while the new peak for DHA at 6.4 min emerged and enhanced gradually (Additional file 1: Fig. S8), indicating the dissociation of disulfide linkage and the release of DHA. In comparison, drug release from DHA_2_-C6 dimer was quite slow under the same condition, and only approximately 10% of DHA released even after 48 h treatment (Fig. 2B). In addition, we further investigated the oxidation responsiveness of these dimers. Similar to reduction responsiveness, DHA_2_-SS also exhibited sensitive oxidation responsiveness. As presented in Fig. 2D, DHA_2_-SS could release about 60% DHA after 48 h of H_2_O_2_ treatment (10 mM), while negligible degradation was detected for DHA_2_-C6 at the same time (Fig. 2E). The release curves of DHA over time in the presence of DTT and H_2_O_2_ are revealed in Fig. 2C, F, respectively. These results validate the redox responsiveness of disulfide bond linker and the controlled release of DHA. The proposed degradation mechanism of DHA_2_-SS dimers initiated by DTT or H_2_O_2_ are illustrated in Additional file 1: Fig. S9 [23, 24, 26–28]. For DTT triggered drug release, because of − SH of DTT attacks, a sulfhydryl − disulfide bond exchange reaction occurs, and the generated thiol groups could facilitate the hydrolysis of the adjacent ester bond and the release of DHA from prodrugs. For H_2_O_2_ triggered drug release, the disulfide bond of DHA_2_-SS could be oxidized to hydrophilic sulfoxide or sulphone upon exposure to H_2_O_2_, leading to hydrolysis of the adjacent ester bond and subsequent release of DHA.

### Cellular uptake and in vitro cytotoxicity of DHA dimeric NPs

**Fig. 3:**
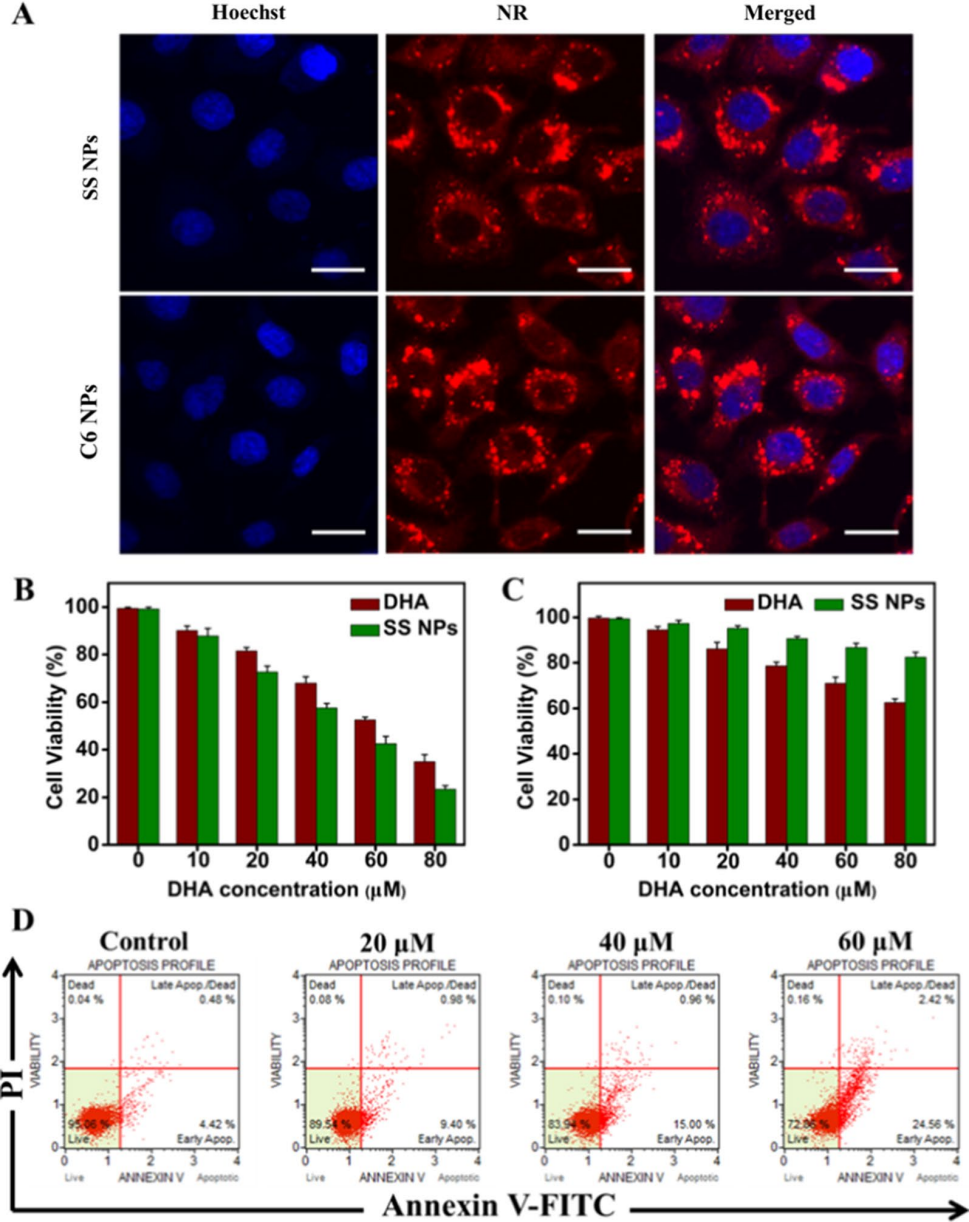
(**A**) CLSM images of HepG2 cells incubated with SS and C6 NPs at 37 °C for 4 h. Scale bars, 20 μm. In vitro cytotoxicity against (**B**) HepG2 cells and (**C**) HL-7702 cells of free DHA and SS NPs at different concentrations after incubation for 48 h. (**D**) Flow cytometry data for apoptosis in HepG2 cells treated with different concentrations of SS NPs

Human hepatoma HepG2 cells were used to study the cellular internalization of these NPs via confocal laser scanning microscopy (CLSM). The fluorescent dye nile red (NR) was utilized as a marker and encapsulated into the NPs by coassembly with DHA dimer, and the blue fluorescence of Hoechst 33258 was employed to localize the cell nucleus. As exhibited in Fig. 3A, the strong red fluorescence signals principally distributed in the cytoplasm, suggesting the efficient internalization of DHA dimeric NPs. And we could also observe that the fluorescence intensity increased obviously from 0.5 h to 4 h, implying that endocytosis continued in a time-dependent manner (Additional file 1: Fig. S10). However, when the incubation time was extended to 8 h, the fluorescence intensity of SS NPs decreased significantly, indicating the dissociation of disulfide bond and the release of DHA, whereas C6 NPs still displayed strong fluorescence signal because of the insensitive responsiveness of C6 linker to the tumor redox microenvironment.

Next, the cytotoxicity of DHA dimeric NPs was evaluated against human HepG2 and HeLa cells through a standard MTT assay. As shown in Fig. 3B, both free DHA and SS NPs showed efficient suppression of HepG2 viability in concentration dependent manner for 48 h, and SS NPs exhibited higher cytotoxicity than free DHA. The cell viability of SS NPs was less than 30%, while it was approximately 40% for free DHA at the equivalent DHA concentration. This result may be attributed to the enhanced cellular uptake of NPs into tumor cells and the rapid release of DHA. And SS NPs could quickly release active DHA once in living cells because of the responsiveness of disulfide bond in redox environment. However, C6 NPs exhibited apparent lower cytotoxicity compared with SS NPs (Additional file 1: Fig. S11). Similarly, SS NPs still possessed the strongest cellular toxicity towards HeLa cells (Additional file 1: Fig. S12). In addition, we evaluated the cytotoxicity of SS NPs toward normal hepatocytes (HL-7702), and also compared its toxicity on HepG2, HeLa and HL-7702 cells. As revealed in Fig. 3C, SS NPs exhibited no significant cellular toxicity against HL-7702 cells, and enhanced cytotoxicity against these two kinds of tumor cells (Additional file 1: Fig. S13), indicating the selectivity of DHA_2_-SS prodrug towards tumor cells. This result is ascribed to the different redox conditions in normal cells and tumor cells.

To further understand the contribution of SS NPs on apoptosis, we stained HepG2 cells with Annexin V-FITC and PI, and analyzed them by flow cytometry. As presented in Fig. 3D, the ratio of early apoptotic cells was 9.40%, 15.00%, and 24.56%, respectively, as the drug concentration increases (20, 40 and 60 μM), validating the cell apoptosis in a concentration-dependent manner. Additionally, we also examined the nuclear morphological changes by CLSM. The cell nucleus emerged as a homogeneous blue chromatin with an organized structure in normal cells, whereas the cells incubated with SS NPs displayed representative morphological changes (Additional file 1: Fig. S14), including intense fluorescent spots, nuclear pyknosis, and extensive blebbing, further verifying the apoptosis of tumor cells.

### Antitumor mechanism of SS NPs

**Fig. 4:**
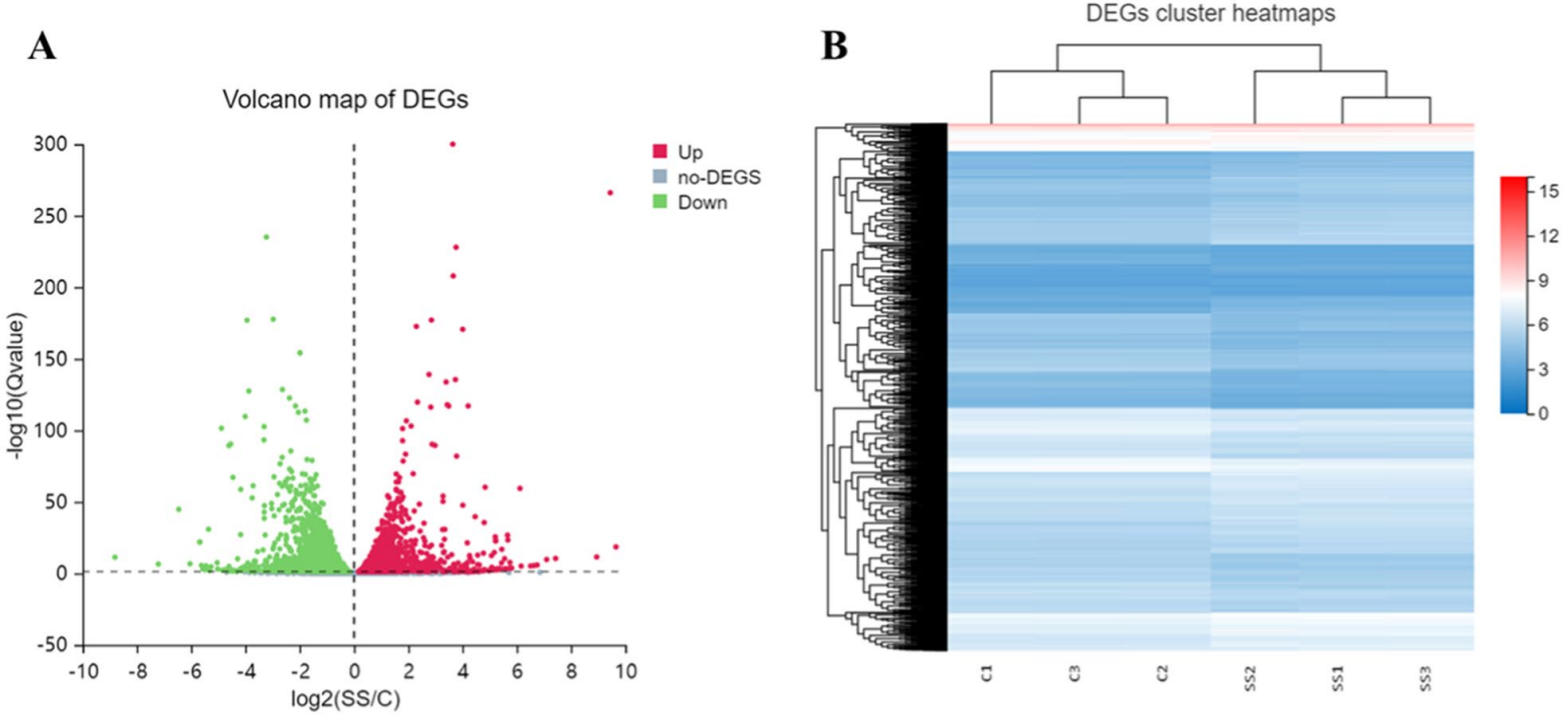
**A** Volcano plots to determine DEGs of SS NPs treatment group and control group. The x-axis represents the log 2.0-fold changes (FCs) of genes and the y-axis represents the − log^10^ of the *p*-values for the various condition pairs. Each dot represents a gene. The gray points represent a non-statistically significant difference in gene expression. The red field represents the upregulated genes and the blue field represents the downregulated genes. **B** Heat map displaying the overview of the differentially expressed genes induced by SS NPs treatment

To elucidate the mechanism of SS NPs in inhibiting tumor cell proliferation and inducing apoptosis, RNA sequencing (RNA-seq) technology was applied to collect the gene expression [50]. The total RNA extracted from SS NPs treatment group (SS) and the control group (C) have been analyzed for quality and integrity by utilizing formaldehyde agarose gel electrophoresis. The results verified that the obtained RNA was intact, undegraded, and suitable for RNA-seq analysis. Meanwhile, principal component analysis (PCA) of samples was performed on the complete dataset [51], which can display changes of overall gene expression. As shown in the PCA results (Additional file 1: Fig. S15), there were two clusters, verifying a distinct directionality between SS NPs treatment and the control groups based on the similarity of gene expression. Subsequently, the differentially expressed genes (DEGs) induced by SS NPs have been identified and described by volcano plots and heatmaps. After comparing with the untreated control group (log_2_ fold-change ≥ 2.0 and adjusted *P* value < 0.05), we distinguished 6546 DEGs, including 3288 up-regulated and 3258 down-regulated expression genes (Fig. 4A, B). The above results imply that SS NPs play an important role on gene expression of the treated tumor cells.

**Fig. 5:**
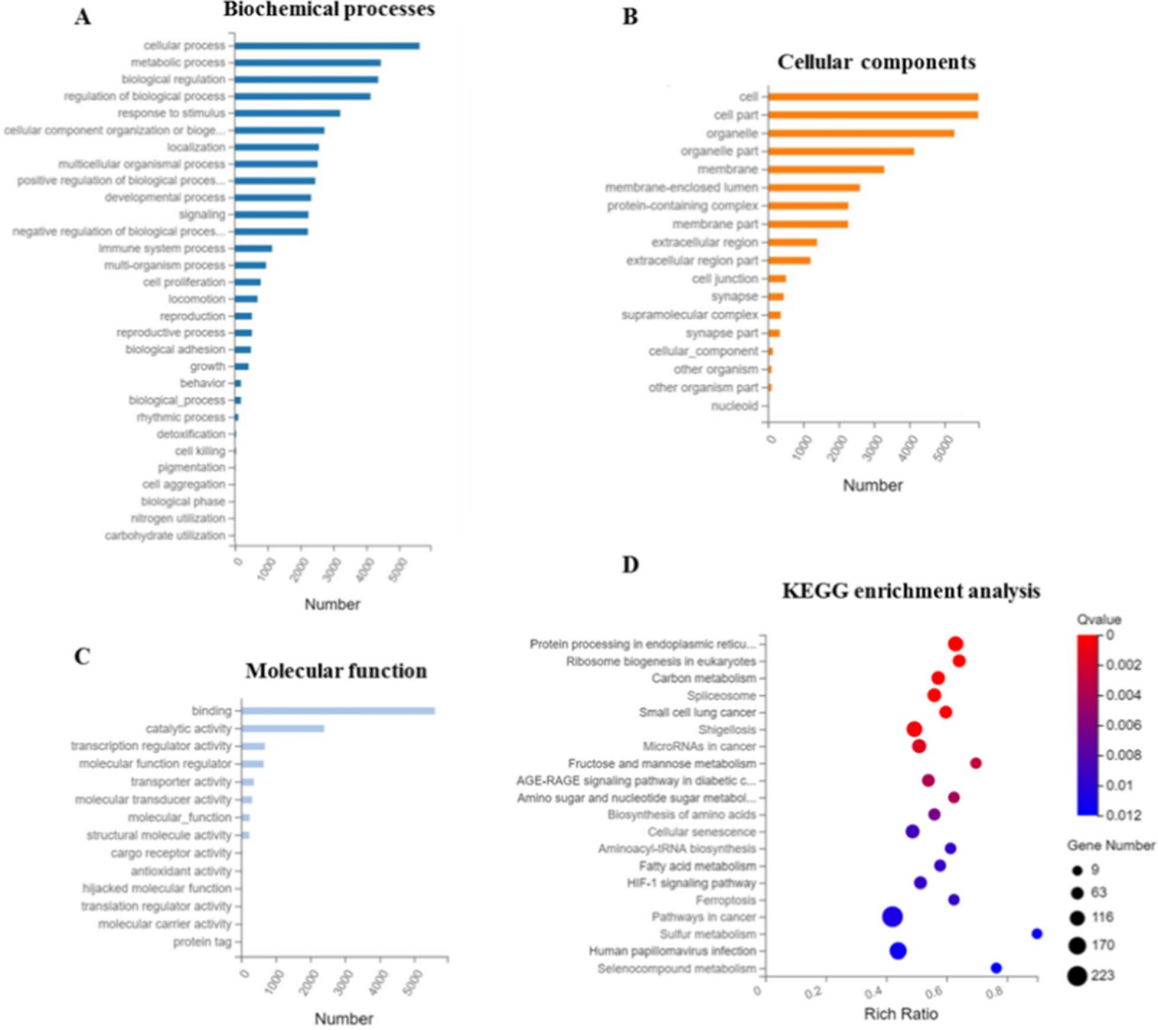
GO and KEGG pathway functional enrichment analysis of DEGs in SS NPs treated HepG2 cells. **A** Biochemical processes. **B** Cellular components. **C** Molecular function. **D** KEGG pathway functional enrichment of DEGs

According to the RNA-seq data, we performed the Gene Ontology (GO) and Kyoto Encyclopedia of Genes and Genomes (KEGG) pathway analysis for 6546 differentially expressed genes. Three domains were involved: cellular components (CC), biological processes (BP) and molecular function (MF). In the BP domain, a large proportion of genes were associated with cellular process, metabolic process, biological regulation (Fig. 5A). For the CC domain, the enriched genes covered cell, cell part, organelle, membrane and so forth (Fig. 5B). Binding, catalytic activity, transcription regulator activity and molecular function regulator were primarily influenced for the MF domain (Fig. 5C).

On the basis of the DEGs results, we also executed KEGG functional enrichment analysis and pathway classification. The KEGG functional enrichment analysis result (Fig. 5D) demonstrated that multiple pathways have been affected by DHA_2_-SS NPs, and the signaling pathways of carbon metabolism, biosynthesis of amino acids, fatty acid metabolism, HIF-1 signaling pathway and pathways in cancer were among the top 20 pathways. On the other hand, KEGG pathway classification results (Additional file 1: Fig. S16) displayed that there were six branches for KEGG pathways, including cellular processes, environmental information processing, genetic information processing, human diseases, metabolism, and organismal systems. And the DEGs in HepG2 cells after SS NPs treatment were mainly enriched in PI3K-Akt signaling pathway, MAPK signaling pathway and pathways in cancer.

**Fig. 6:**
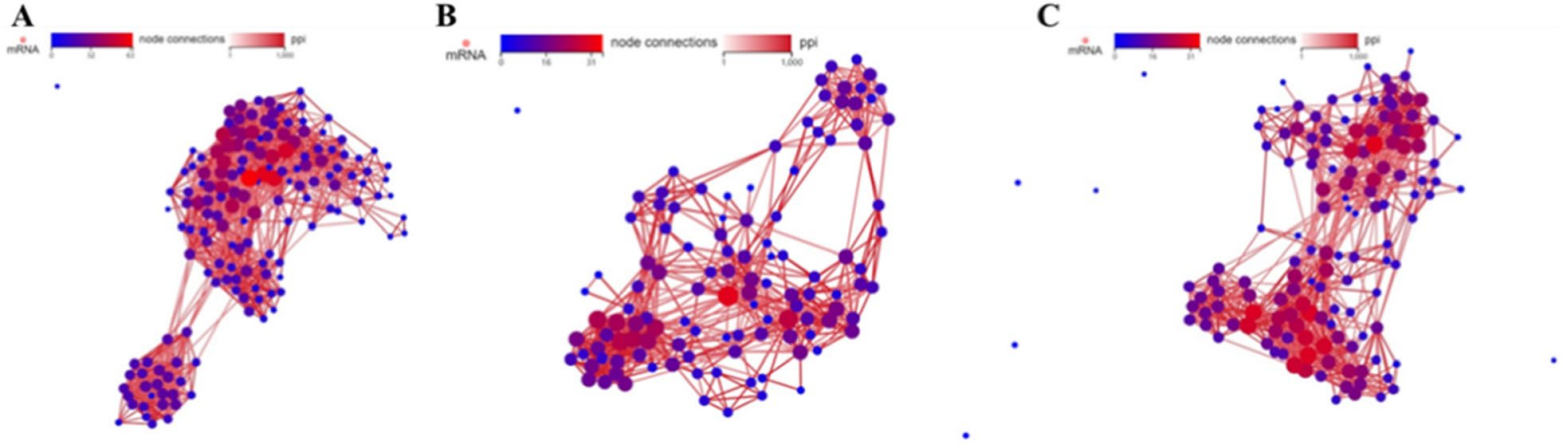
PPI network of the DEGs correlation with (**A**) carbohydrates, (**B**) amino acids and (**C**) lipids metabolism

From GO and pathway analysis results, we discovered that the metabolic process was involved in the inhibition of tumor cells proliferation, thus we carried out network statistical analysis on the protein–protein interaction (PPI) of these genes correlated with metabolism. As shown in Fig. 6, the metabolism of carbohydrates, amino acids and lipids have been remarkably regulated in the cells after SS NPs treatment, indicating that SS NPs could induce metabolic reprogramming in tumor cells. Therefore, we concentrated on the metabolism reprogramming of tumor cells after being treated with SS NPs.

**Fig. 7:**
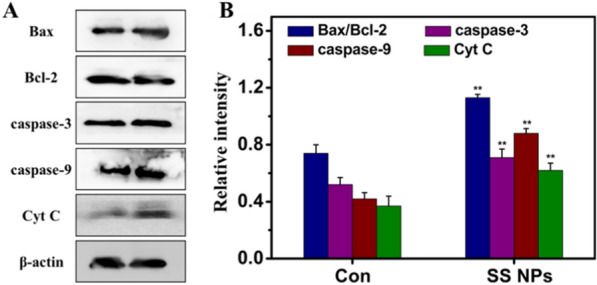
Effects of SS NPs on expression of mitochondrial apoptosis pathway relative proteins. **A** Protein expression was analyzed using western blotting and (**B**) quantified in relation to β-actin. Densitometric values were normalized by β-actin and expressed as mean ± SD, n = 3. Statistical significance: *p < 0.05, and **p < 0.01

Apoptosis is a highly regulated process of cell death, which is important to maintain the inherent stability of multicellular organisms, and involves a variety of signal pathways [52–54]. The Bcl-2 protein family contains pro-apoptotic and anti-apoptotic regulators of programmed cell death/apoptosis, and plays a dominant role in regulating cell apoptosis. In this family, Bax gene, a pro-apoptotic member, can form heterodimers with Bcl-2 protein, and the ratio of Bax/Bcl-2 could determine the sensitivity of cells to apoptosis. The activation of Bax can release cytochrome c (Cyt C), and Cyt C activates caspase-9 and downstream caspase-3 through a cascade reaction to promote cell apoptosis, whereas the anti-apoptotic Bcl-2 operates in the opposite way. Therefore, we detected expression of Bax, Bcl-2, cleaved caspase-3, cleaved caspase-9 and Cyt C by western blotting. As displayed in Fig. 7, the expression levels of Bax, caspase-3, caspase-9, and Cyt C were obviously increased, and the expression levels of Bcl-2 was reduced compared to the control group. The above results demonstrate that mitochondrial apoptosis pathway is involved in the apoptosis of HepG2 cells induced by SS NPs.

**Fig. 8:**
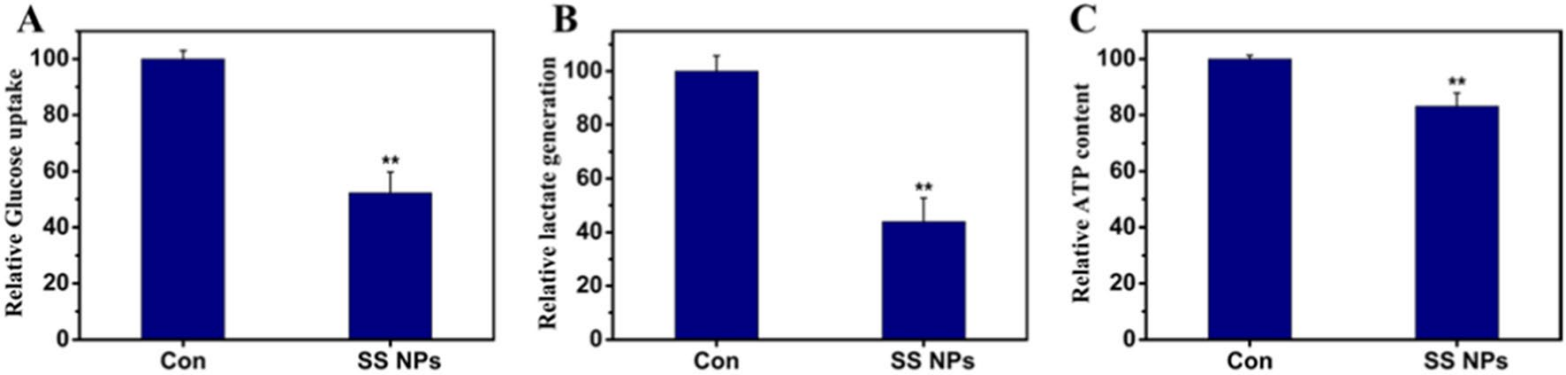
SS NPs suppress glycolysis level in HepG2 cells. **A** After HepG2 cells were treated with SS NPs, glucose content in the culture media was immediately tested, and glucose uptake was calculated. **B** Lactic acid product was detected. **C** ATP content was detected through bioluminescence assay. Data arepresented as mean ± SD, n = 3. Statistical significance: *p < 0.05, and **p < 0.01

As we known, tumor cells generally reveal aberrant metabolism due to metabolic reprogramming [55]. As a hallmark of tumor cells, the Warburg effect means that tumor cells rely heavily on glycolysis for energy, rather than oxygen [56–58]. To investigate whether SS NPs treatment could suppress the glycolysis of tumor cells, the glucose uptake, contents of lactic acid and ATP products of HepG2 cells treated with SS NPs were detected. As shown in Fig. 8, the glucose uptake was decreased, and the contents of intracellular ATP and extracellular lactic acid were also declined, indicating that SS NPs may inhibit glycolysis of tumor cells.

**Fig. 9:**
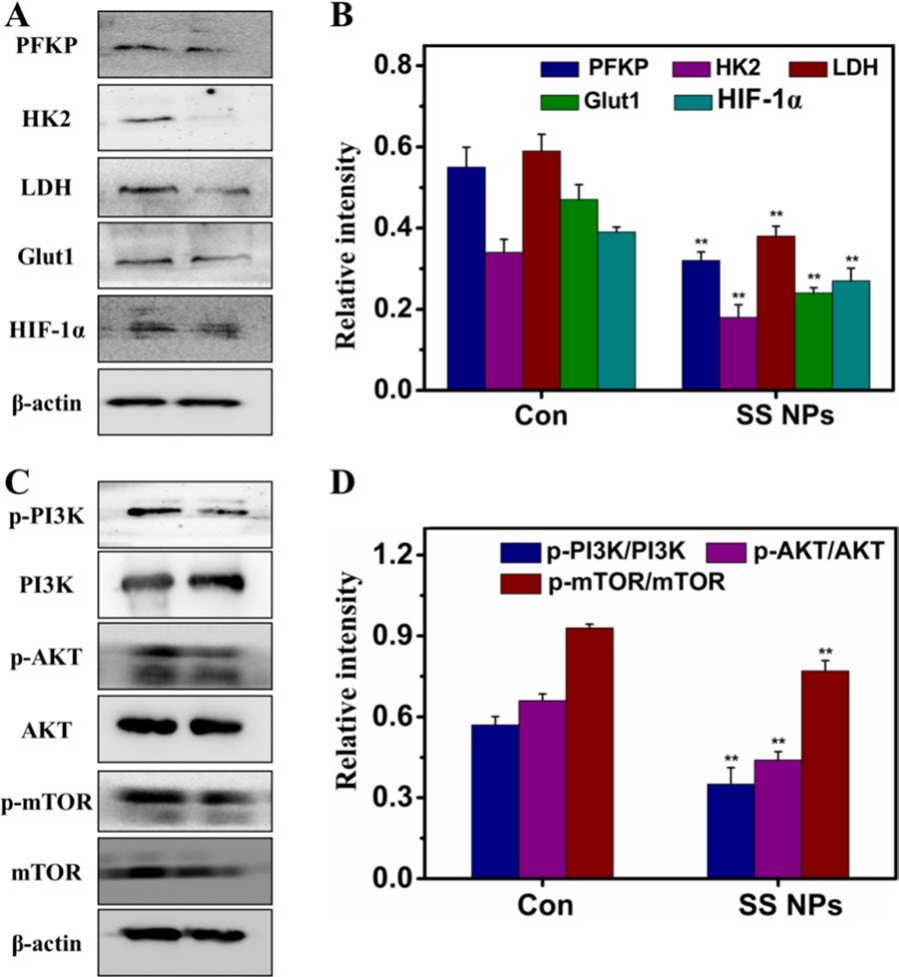
Effects of SS NPs on expression of PI3K/AKT/HIF-1α signaling pathway relative proteins. **A** Protein expression of glycolytic enzymes were analyzed using western blotting and (**B**) quantified in relation to β-actin. **C** Protein expression of PI3K/AKT pathway were analyzed and (**D**) quantified in relation to β-actin. Densitometric values were normalized by β-actin and expressed as mean ± SD, n = 3. Statistical significance: *p < 0.05, and **p < 0.01

Cancer metabolic reprogramming is regulated by multiple pathways, which includes the PI3K-AKT signaling pathway [59]. The activated PI3K-AKT can promote the transition to aerobic glycolysis, and AKT could result in the phosphorylation of some important downstream targets, such as Bcl-2 apoptosis-related family, and mammalian target of rapamycin (mTOR), to protect cells from apoptosis. Meanwhile, this pathway also could regulate HIF-1α through mTOR, and activated HIF-1α is related to the up-regulation of glucose transporters (Gluts) and glycolytic enzymes. To understand whether the activation of PI3K/AKT and HIF-1α were involved in metabolic reprogramming of tumor cells treated by SS NPs, we detected the related protein expression. As displayed in Fig. 9, SS NPs treatment could decrease the expression of glycolytic enzymes, such as PFKP, HK2, LDH, Glut1. The ratio of p-PI3K/PI3K, p-AKT/AKT, p-mTOR/mTOR and HIF-1α were also reduced accordingly. Collectively, these findings confirm that SS NPs could induce apoptosis and suppress glycolysis by regulating the PI3K/AKT/HIF-1α signaling pathway.

### In vivo antitumor efficacy of DHA dimeric NPs

**Fig. 10:**
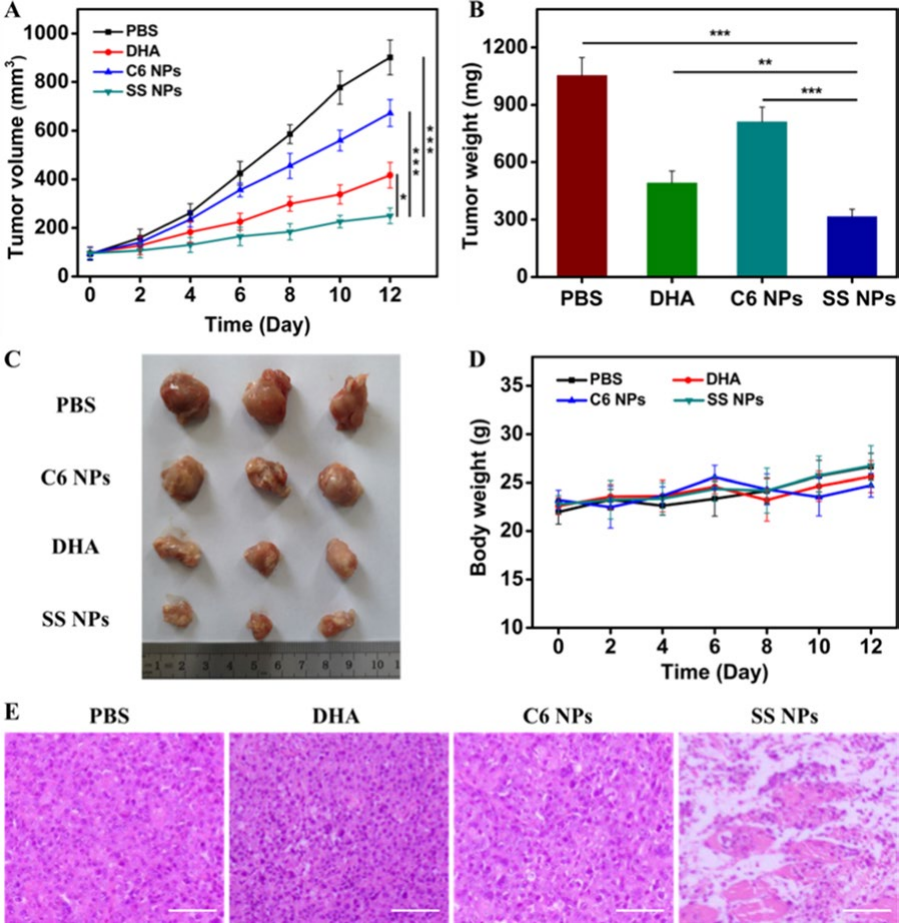
In vivo antitumor efficacy evaluation. **A** Tumor growth curves and (**B**) tumor weights of H22 tumor-bearing mice after different treatments. **C** Photographs of excised tumors after the last treatment. **D** Body weight curves of tumor-bearing mice in each group during treatments. **E** H&E-stained images of tumor slices collected from mice after different treatments. Scale bars, 50 μm. Data are expressed as mean ± SD (*n* = 3). Statistical significance: **p < 0.01, and ***p < 0.001

We further evaluated the anti-cancer activity of SS NPs on H22 tumor-bearing Kunming mice. Mice bearing the tumors were randomly divided into 4 groups with different treatments: PBS, free DHA, C6 NPs, and SS NPs, and injected intravenously at equivalent DHA doses every second day. As illustrated in Fig. 10A, DHA treatment exhibited a moderate inhibitory effect on tumor growth compared with the control group, which is mainly owing to its intrinsic toxicity. Notably, SS NPs exhibited evident antitumor activity, which is more potent than free DHA group. The improved therapeutic efficacy of SS NPs should be ascribed to the multiple advantages of nanoparticle formulations, including enhanced tumor accumulation, effective endocytosis, and rapid drug release in tumor sites. Unsurprisingly, C6 NPs group displayed the weakest tumor growth inhibition effect in these treatment groups on account of the insensitivity of C6 linker to redox microenvironment. What’s more, the tumor weight (Fig. 10B) and the photographs of resected tumors (Fig. 10C) visually demonstrated the greatest tumor inhibition efficacy obtained by SS NPs, further validating the enhanced antitumor effect of disulfide-bond bridged prodrug nanoparticles. In addition, all mice had no significant weight fluctuation during the whole treatment period (Fig. 10D), and there was also no detectable histological damage observed after SS NPs treatment from the hematoxylin and eosin (H&E) stained tissue sections of major organs (heart, liver, spleen, lung, and kidney) (Additional file 1: Fig. S17). The above results validate SS NPs at current doses possess favorable biosafety and ignorable systemic toxicity. The H&E staining of tumor slices revealed that SS NPs group exhibited the most severe cellular damage compared with the control group. The tumor cells after SS NPs treatment shrank largely and the tumor tissue significantly decreased. (Fig. 10E). All of the results substantiate that DHA_2_-SS NPs could be safely used for the in vivo treatment, and possess better treatment effects in contrast with free DHA.

## Conclusion

In conclusion, a DHA dimeric nanoprodrug using disulfide bond as linkage (DHA_2_-SS) was obtained. The SS NPs not only possess favorable stability and high drug content of 90.6 wt%, but also can respond to the tumor redox microenvironment, thus resulting in the effective release of DHA for chemotherapy. Importantly, both in vitro and in vivo therapy experiments indicate these obtained SS NPs have efficient endocytosis, potent cytotoxicity and enhanced antitumor efficacy in contrast with free DHA. RNA sequencing and bioinformatics analysis demonstrate that SS NPs could induce apoptosis via the intrinsic mitochondrial apoptosis pathway, as well as inhibit glycolysis through PI3K/AKT/HIF-1α signaling pathway. Our finding provides a reference for the rational design of responsive prodrug nanoparticles, and could potentially spur the similar study of Chinese medicine and related natural active ingredients.

## Supplementary Information

**Additional file 1: Fig. S1.** The synthetic route of DHA_2_-SS and DHA_2_-C6. **Fig. S2.** ^1^H NMR spectra of (A) DHA, (B) DHA_2_-C6 and (C) DHA_2_-SS in CDCl_3_. **Fig. S3.** Mass spectrum of DHA_2_-SS. **Fig. S4.** Mass spectrum of DHA_2_-C6. **Fig. S5.** Photographs of SS and C6 NPs which were (a) freshly made, (b) 7 days after being immersed in water and (c) 24 h after being immersed in PBS with FBS (10%). **Fig. S6.** FTIR spectra of SS and C6 NPs which were (a) freshly made and (b) 7 days after being immersed in water. **Fig. S7.** TEM images of SS NPs after being immersed in (A) PBS (pH 7.4) and (B) PBS with FBS (10%) for 24 h. TEM images of C6 NPs after being immersed in (C) PBS (pH 7.4) and (D) PBS with FBS (10%) for 24 h. **Fig. S8.** HPLC spectrum of DHA. **Fig. S9.** Schematic illustration of redox-responsive drug release from DHA_2_-SS triggered by DTT/H_2_O_2_. **Fig. S10.** CLSM images of HepG2 cells incubated with (A) SS NPs and (B) C6 NPs at 37 °C for different times. Scale bars, 20 μm. **Fig. S11.** Cell viabilities of C6 and SS NPs against HepG2 cells at different concentrations after incubation for 48 h. **Fig. S12.** Cell viabilities of free DHA, C6 and SS NPs against HeLa cells at different concentrations after incubation for 48 h. **Fig. S13.** Cell viabilities of SS NPs against HL-7702, HeLa and HepG2 cells at different concentrations after incubation for 48 h. **Fig. S14.** Morphological apoptosis by staining with Hoechst 33258 in HepG2 cells treated with different concentrations of SS NPs. **Fig. S15.** Principal component analysis (PCA) of HepG2 cells based on untreated control group (C) and SS NPs treatment group (SS). **Fig. S16.** KEGG pathway classification of differential expressed genes (DEGs). X axis represents number of DEGs, Y axis represents functional classification of KEGG. **Fig. S17.** H&E staining of the major organs (heart, liver, spleen, lung and kidney) of mice with H22 tumor xenografts after different treatments. Scale bars: 100 μm.

## Data Availability

All data generated or analysed during this study are included in this article and its Additional file.
