# Supplementary material for: A redox-responsive dihydroartemisinin dimeric nanoprodrug for enhanced antitumor activity
Source: J Nanobiotechnology. 2021 Dec 20;19:441. doi: 10.1186/s12951-021-01200-z (PMC8686335; doi:10.1186/s12951-021-01200-z)
Supplement: Supplementary file 1 — Additional file 1: Fig. S1. The synthetic route of DHA2-SS and DHA2-C6. Fig. S2. 1H NMR spectra of (A) DHA, (B) DHA2-C6 and (C) DHA2-SS in CDCl3. Fig. S3. Mass spectrum of DHA2-SS. Fig. S4. Mass spectrum of DHA2-C6. Fig. S5. Photographs of SS and C6 NPs which were (a) freshly made, (b) 7 days after being immersed in water and (c) 24 h after being immersed in PBS with FBS (10%). Fig. S6. FTIR spectra of SS and C6 NPs which were (a) freshly made and (b) 7 days after being immersed in water. Fig. S7. TEM images of SS NPs after being immersed in (A) PBS (pH 7.4) and (B) PBS with FBS (10%) for 24 h. TEM images of C6 NPs after being immersed in (C) PBS (pH 7.4) and (D) PBS with FBS (10%) for 24 h. Fig. S8. HPLC spectrum of DHA. Fig. S9. Schematic illustration of redox-responsive drug release from DHA2-SS triggered by DTT/H2O2. Fig. S10. CLSM images of HepG2 cells incubated with (A) SS NPs and (B) C6 NPs at 37 °C for different times. Scale bars, 20 μm. Fig. S11. Cell viabilities of C6 and SS NPs against HepG2 cells at different concentrations after incubation for 48 h. Fig. S12. Cell viabilities of free DHA, C6 and SS NPs against HeLa cells at different concentrations after incubation for 48 h. Fig. S13. Cell viabilities of SS NPs against HL-7702, HeLa and HepG2 cells at different concentrations after incubation for 48 h. Fig. S14. Morphological apoptosis by staining with Hoechst 33258 in HepG2 cells treated with different concentrations of SS NPs. Fig. S15. Principal component analysis (PCA) of HepG2 cells based on untreated control group (C) and SS NPs treatment group (SS). Fig. S16. KEGG pathway classification of differential expressed genes (DEGs). X axis represents number of DEGs, Y axis represents functional classification of KEGG. Fig. S17. H&E staining of the major organs (heart, liver, spleen, lung and kidney) of mice with H22 tumor xenografts after different treatments. Scale bars: 100 μm. [file 12951_2021_1200_MOESM1_ESM.docx]

**Supplementary Information
A redox-responsive dihydroartemisinin dimeric nanoprodrug for enhanced antitumor activity**

Yawei Li^1^, Qing Pei^2^, Baiji Cui^1^, Hongmei Zhang^1^, Liu Han^1^, Wenqing Li^1^, Wenhe Zhu^1*^, Xianmin Feng^1*^, and Zhigang Xie^2*^

^1^ Jilin Medical University, Jilin 132013, P. R. China

^2^ State Key Laboratory of Polymer Physics and Chemistry, Changchun Institute of Applied Chemistry, Chinese Academy of Sciences, Changchun, 130022, P. R. China

^*^Corresponding author [xiez@ciac.ac.cn](mailto:xiez@ciac.ac.cn); [huolizwh@163.com](mailto:huolizwh@163.com); fengxianmin28@163.com

**Experimental section**

**Materials**

Dihydroartemisinin (DHA) was purchased from Shanghai Aladdin Biochemical Technology Co. Ltd., 1-Ethyl-3- (3-dimethylaminopropyl) carbodiimide hydrochloride (EDC·HCl, GL Biochem), 4-dimethylaminopyridine (DMAP, Aladdin) and 2,2'-disulfanediyldiacetic acid (TCI Shanghai) were used as received. Chloroform-d (CDCl_3_) was purchased from Qingdao Tenglong Weibo Technology Co. Ltd. Dithiothreitol (DTT) were purchased from Aladdin Co., Ltd. Annexin V-FITC apoptosis detection kit was purchased from Shanghai Beyotime Biotechnology Co., Ltd. Ultrapure water was prepared from a Milli-Q system (Millipore, USA).

**Synthesis of DHA_2_-SS**

DHA (0.16 mmol) was dissolved in dichloromethane (CH_2_Cl_2_), and then 2,2'-disulfanediyldiacetic acid (0.089 mmol), EDC·HCl (0.35 mmol) and DMAP (0.018 mmol) were added sequentially. After stirring for 1 h at ambient temperature, additional EDC·HCl (0.17 mmol) and DMAP (0.018 mmol) were added, and reaction was continued for another 24 h. The reaction product was purified using silica gel column chromatography with CH_2_Cl_2_ and ethyl acetate to give DHA_2_-SS. The synthesis process of DHA_2_-C6 was similar to that of DHA_2_-SS, so it is not described in detail here. All above reaction yields were > 90%.

**Preparation of NPs**

For the preparation of SS and C6 NPs, 4 mg of the corresponding DHA dimer was dissolved in 4 mL of tetrahydrofuran. This solution was added dropwise into 10 mL of ultrapure water in ten minutes, then stirred for overnight to volatilize tetrahydrofuran. At last the crude product was dialyzed against water with a 3500D dialysis bag to removal residual tetrahydrofuran. Finally, DHA dimeric prodrug NPs were obtained.

**In vitro stability of NPs**

To investigate the in vitro stability, DHA dimer NPs were incubated in a solution of PBS (pH 7.4) containing 10% fetal bovine serum (FBS) at temperature of 37 ^o^C for different times. The changes of particle size and size distribution was monitored by DLS.

**DTT or H_2_O_2_-triggered hydrolysis of DHA dimer**

The in vitro degradation of two different linkers and the release of DHA from DHA dimer in vitro were investigated by incubating DHA dimer (50 μg) in 500 μL release media (acetonitrile/PBS, v/v = 1/1, pH 7.4) with DTT or H_2_O_2_ at 37 ^o^C. After a certain period of incubation, 500 μL of relase media was extracted and mixed with 500 μL of acetonitrile. Then the degradation behavior of dimers was detected by HPLC.

**Cellular uptake**

Cellular uptakes by HepG2 cells were examined using a confocal laser scanning microscope (CLSM). Cells were seeded in 6-well culture plates (a sterile cover slip was put in each well) at a density of 5×10^4^ cells per well and allowed to adhere for 24 h. After that, cells were incubated with DHA dimeric NPs (20 µM) for 0.5 h, 2 h, 4 h, or 8 h at 37 ^o^C. Subsequently, the supernatant was carefully removed and the cells were washed three times with PBS. Subsequently, the cells were ﬁxed with 500 μL of 4% formaldehyde in each well for 20 min at room temperature and washed twice with PBS again. Cells were visualized using blue channel for Hoechst 33258 and red channel for NR under a confocal laser scanning microscope.

**Cytotoxicity test**

The cytotoxicity test was measured *via* MTT assay. HepG2, HeLa or HL-7702 cells harvested in a logarithmic growth phase were seeded in 96-well plates at a density of 10^5^ cells/well and incubated in DMEM for 24 h. The medium was then replaced by DHA or DHA dimeric prodrug NPs, at a final equivalent DHA concentration from 10 to 80 μM for each drug. The incubation was continued for 48 h. Then, 20 μL of MTT solution in PBS with the concentration of 5 mg/mL was added and the plates were incubated for another 4 h at 37 ^o^C, followed by removal of the culture medium containing MTT and addition of 150 μL of DMSO to each well to dissolve the formazan crystals formed. Finally, the plates were shaken for 10 min, and the absorbance of formazan product was measured at 490 nm by a microplate reader. Each data point was an average of three independent experiments.

**Apoptosis**

The apoptosis and necrosis induced by SS NPs were evaluated by ﬂow cytometry. HepG2 cells treated with different concentrations of SS NPs were harvested by centrifugation at 1000 rpm for 5 min, and washed with ice-cold PBS. The cell suspension (100 μL) was centrifuged at 1000 rpm for 5 min. After that, the supernatant was discarded and the pellet was gently resuspended in 195 μL annexin V-FITC binding buffer, and incubated with 5 μL propidium iodide (PI) solution on an ice bath in the dark. After ﬁltration (300 μm), the suspension from each group was analyzed using a flow cytometry.

**Total RNA extraction and mRNA library construction**

Total RNA was extracted from the cells DHA_2_-SS NPs treated or control cells using Trizol (Invitrogen, Carlsbad, CA, USA) according to manual instruction. Total RNA was qualified and quantified using a Nano Drop and Agilent 2100 bioanalyzer (Thermo Fisher Scientific, MA, USA). Oligo(dT)-attached magnetic beads were used to purified mRNA. Purified mRNA was fragmented into small pieces with fragment buffer at appropriate temperature. Then First-strand cDNA was generated using random hexamer-primed reverse transcription, followed by a second-strand cDNA synthesis. Afterwards, A-Tailing Mix and RNA Index Adapters were added by incubating to end repair. The cDNA fragments obtained from previous step were amplified by PCR, and products were purified by Ampure XP Beads, then dissolved in EB solution. The product was validated on the Agilent Technologies 2100 bioanalyzer for quality control. The double stranded PCR products from previous step were heated denatured and circularized by the splint oligo sequence to get the final library. The single strand circle DNA (ssCir DNA) was formatted as the final library. The final library was amplified with phi29 to make DNA nanoball (DNB) which had more than 300 copies of one molecular, DNBs were loaded into the patterned nanoarray and single end 50 bases reads were generated on BGIseq500 platform (BGI-Shenzhen, China).

**Bioinformatics analysis**

The sequencing data was filtered with SOAPnuke (v1.5.2) by removing reads containing sequencing adapter. The clean reads were mapped to the reference genome using HISAT2 (v2.0.4). Bowtie2 (v2.2.5) was applied to align the clean reads to the reference coding gene set，then expression level of gene was calculated by RSEM (v1.2.12). The heatmap was drawn by pheatmap (v1.0.8) according to the gene expression in different samples. Essentially, differential expression analysis was performed using the DESeq2 (v1.4.5) with Q value ≤ 0.05. To take insight to the change of phenotype, GO and KEGG enrichment analysis of annotated different expressed gene was performed by Phyper based on Hypergeometric test. The significant levels of terms and pathways were corrected by Q value with a rigorous threshold (Q value ≤ 0.05) by Bonferroni.

**Western blot and antibodies**

Western blotting was performed using standard methods. After being treated with different concentrations of DHA_2_-SS NPs, the cell pellets were suspended in radio immunoprecipitation assay buffer (150 mM sodium chloride, 50 mM Tris pH 8.0, 1% Triton X-100, 0.5% sodium deoxycholate, 0.1% SDS) supplemented with 10 mM NaF, 1mM Na_3_VO_4_, 5 mM EDTA, 1 mM EGTA, 5 mg/ml leupeptin, 1 mg/mL pepstatin A, 1 mM phenyl methylsulfonyl fluoride, and protease and phosphatase inhibitor for 15 min at 4 ^o^C and centrifuged at 12000 rpm 20 min at 4 ^o^C. Protein concentration of the supernatants was determined using a BCA protein Assay Kit (Beyotime Biotechnology, China). Equal amounts (50 μg) of the proteins were resolved by 12% SDS-polyacrylamide gel electrophoresis gels and transferred to PVDF membranes (Millipore) using a Bio-Rad Trans-blot instrument. Membranes were blocked in 5% milk for 1 h at room temperature following incubation with the indicated primary antibody overnight at 4 °C, washed 3 times with TBS-T buffer, incubated with secondary antibody IgG-HRP at 1:20000 dilutionsin TBS-T buffer. After washing 3 times with TBS-T buffer, the membrane was developed with ECL substrate (Thermo Scientific) and the signal was detected by a BIO-RAD Fluorescent Imager, following quantification by Image Lab software.

The following antibodies were used in this study, all diluted in in TBS-T including 3% milk. Antibodies directed against, P-AKT, AKT, P53, Cleaved Caspase-3, Cleaved Caspase-9, Cyt C, Bax, Bcl-2 were obtained from Abcam (1:1 000 dilution), p-mTOR, mTOR, HIF-1α, Glut1, β-actin were obtained from proteintech (1:5 000 dilution).

**In vivo antitumor test and safety evaluation**

All the experimental procedures to mouse described herein have gained approval from the Ethics Committee of Jilin Medical University and carried out corresponding to the regulation, principles, and guidelines of Chinese law concerning the protection of animal life. Kunming (KM) female mice were obtained from Jilin University and maintained under required conditions. The H22 xenograft tumor models were established by injecting H22 hepatocellular carcinoma cells into the left infra-axillary dermis of the mice. When the tumor grew to a size of ~100 mm^3^, H22 bearing Kunming mice were randomly divided into four groups with 3 mice in each group: PBS, free DHA, C6 NPs, and SS NPs. Mice were administered PBS, free DHA, C6 NPs, and SS NPs with the same dosage of 15 mg/kg DHA *via* tail vein injection once every 2 days, respectively, and the tumor volume and body weight were measured every other day in 12 days. After 12 days of observation and measurement, the mice of four groups were sacrificed and the tumors were excised to intuitionally evaluate the tumor inhibition. At last, main organs (heart, liver, spleen, lung, kidney) and tumor were collected, fixed in 4% paraformaldehyde solution, and then embedded in paraffin, sliced and stained with hematoxylin and eosin (H&E) to evaluate potential toxicity for main organs and apoptosis degree for cancer cells.

**Results**


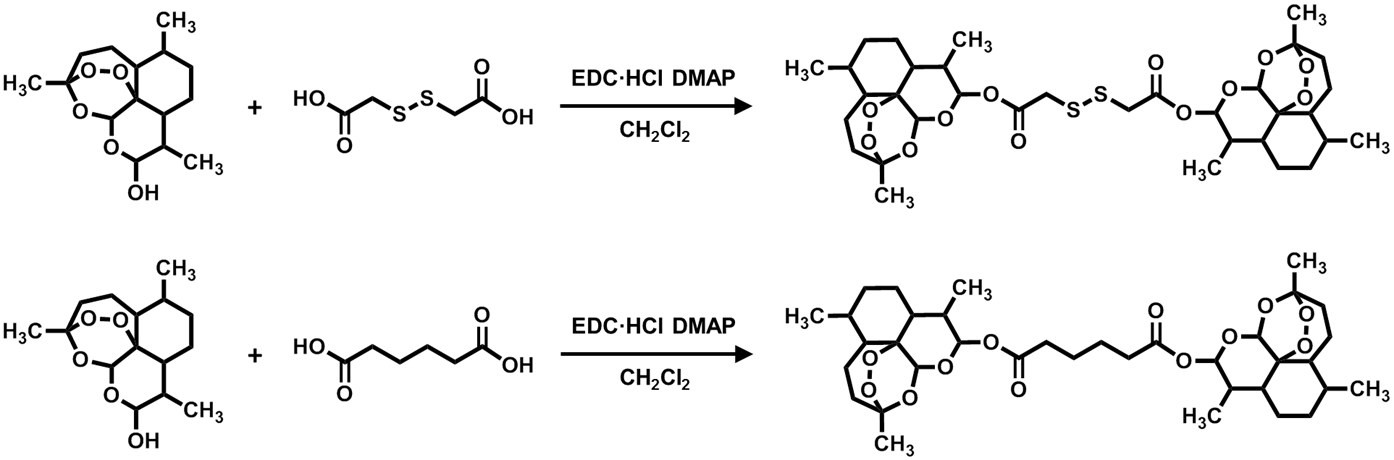


**Fig. S1.** The synthetic route of DHA_2_-SS and DHA_2_-C6.

**
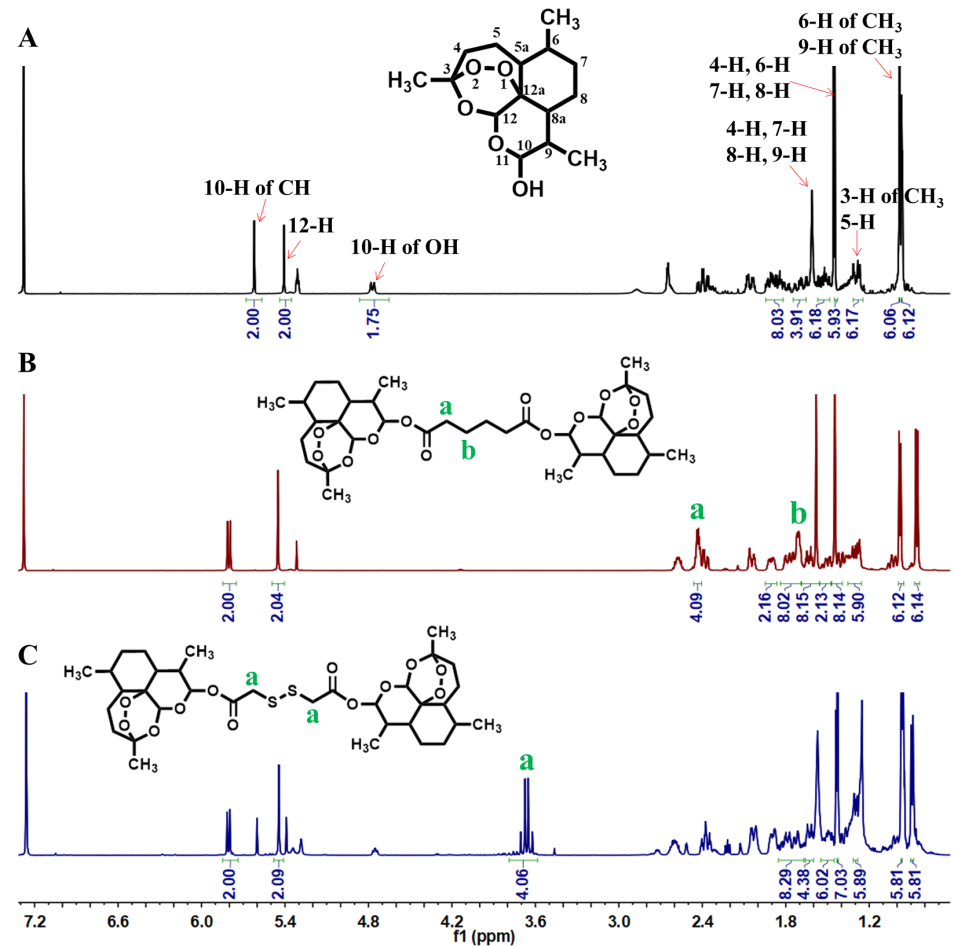
**

**Fig. S2.** ^1^H NMR spectra of (**A**) DHA, (**B**) DHA_2_-C6 and (**C**) DHA_2_-SS in CDCl_3_.


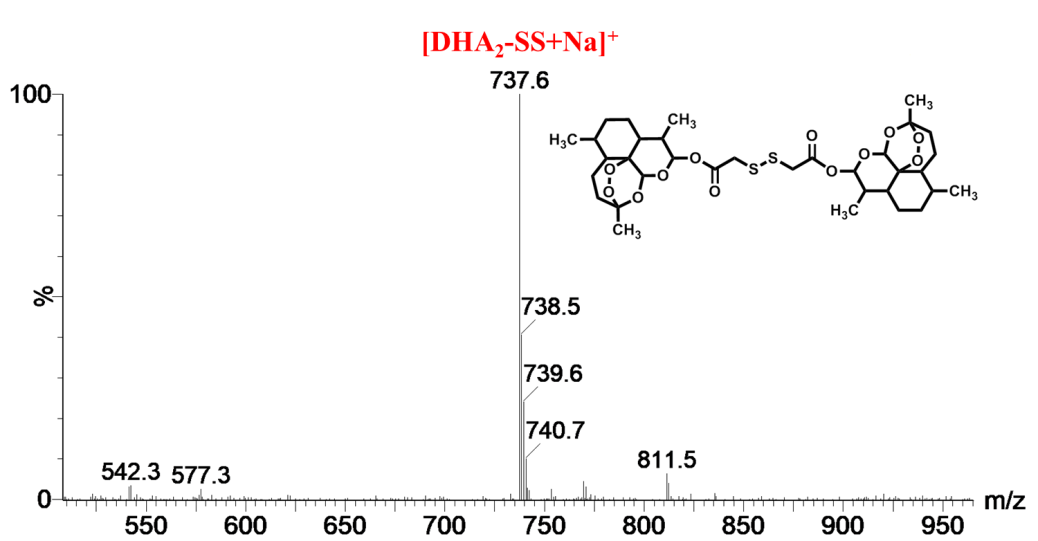


**Fig. S3.** Mass spectrum of DHA_2_-SS.


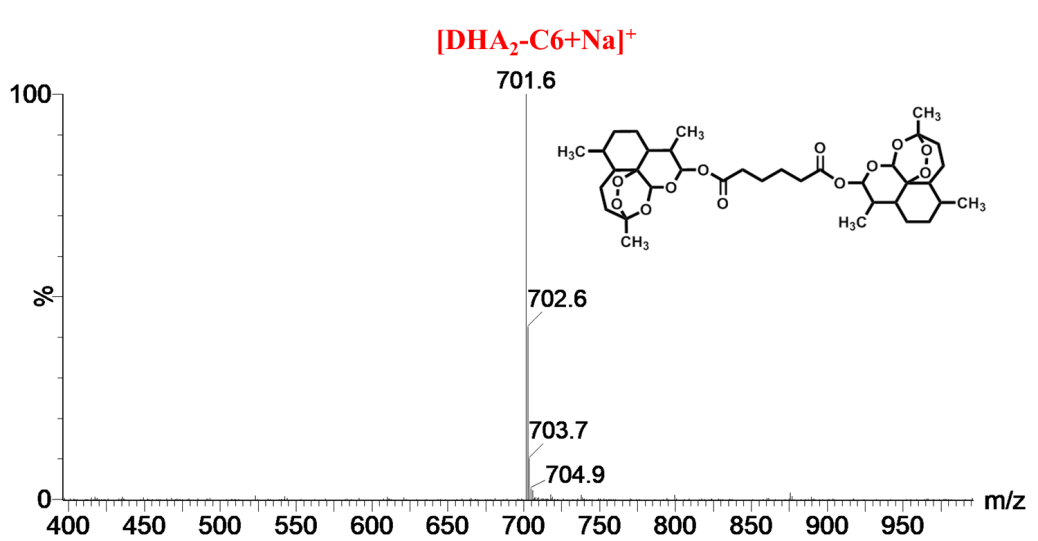


**Fig. S4.** Mass spectrum of DHA_2_-C6.


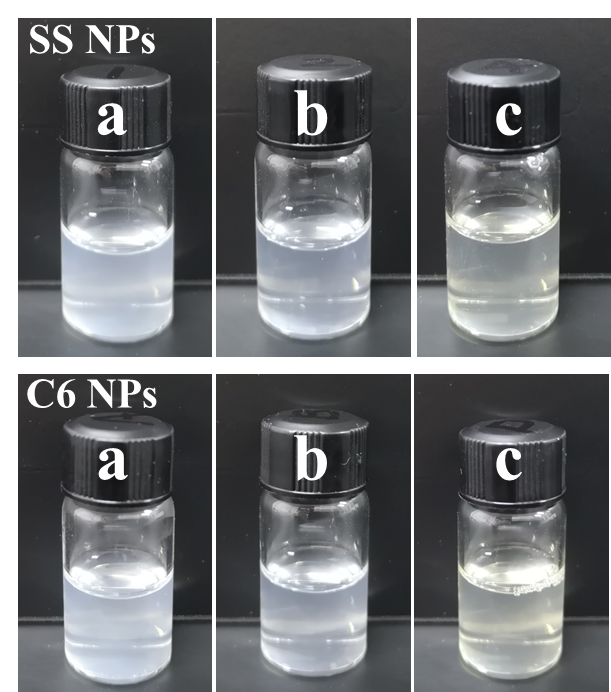


**Fig. S5** Photographs of SS and C6 NPs which were (**a**) freshly made, (**b**) 7 days after being immersed in water and (**c**) 24 h after being immersed in PBS with FBS (10%).


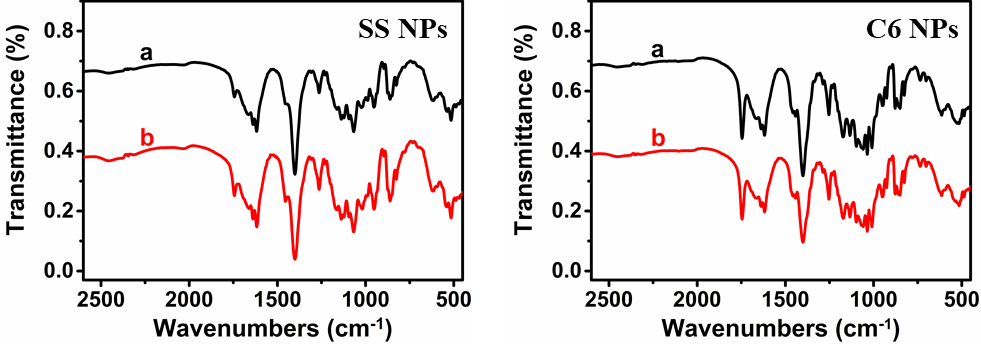


**Fig. S6.** FTIR spectra of SS and C6 NPs which were (**a**) freshly made and (**b**) 7 days after being immersed in water.


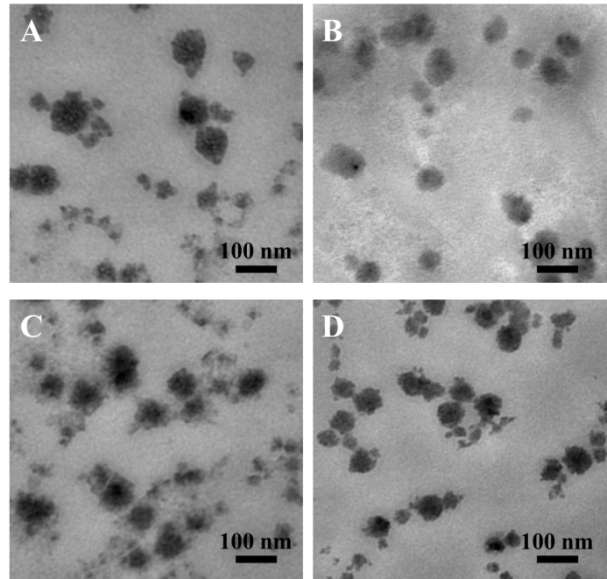


**Fig. S7** TEM images of SS NPs after being immersed in (**A**) PBS (pH 7.4) and (B) PBS with FBS (10%) for 24 h. TEM images of C6 NPs after being immersed in (**C**) PBS (pH 7.4) and (**D**) PBS with FBS (10%) for 24 h.


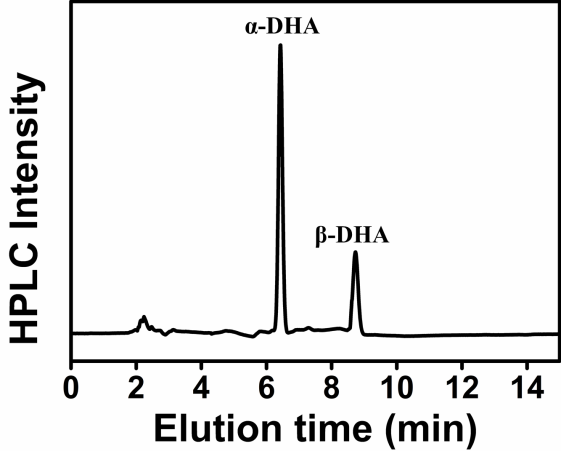


**Fig. S8.** HPLC spectrum of DHA.


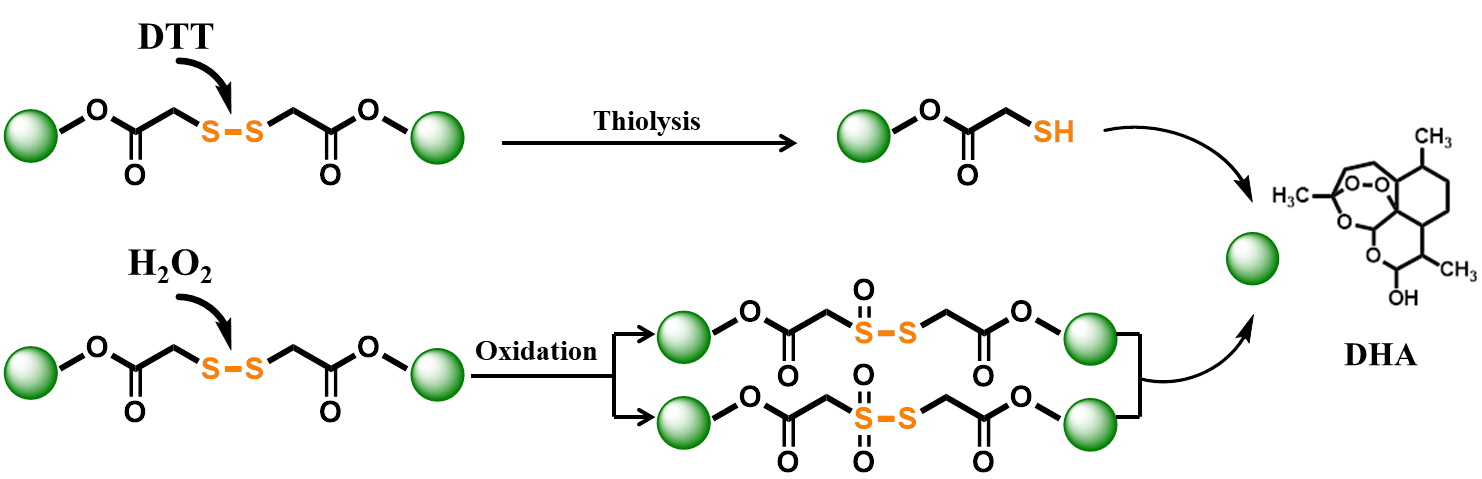


**Fig. S9.** Schematic illustration of redox-responsive drug release from DHA_2_-SS triggered by DTT/H_2_O_2_.


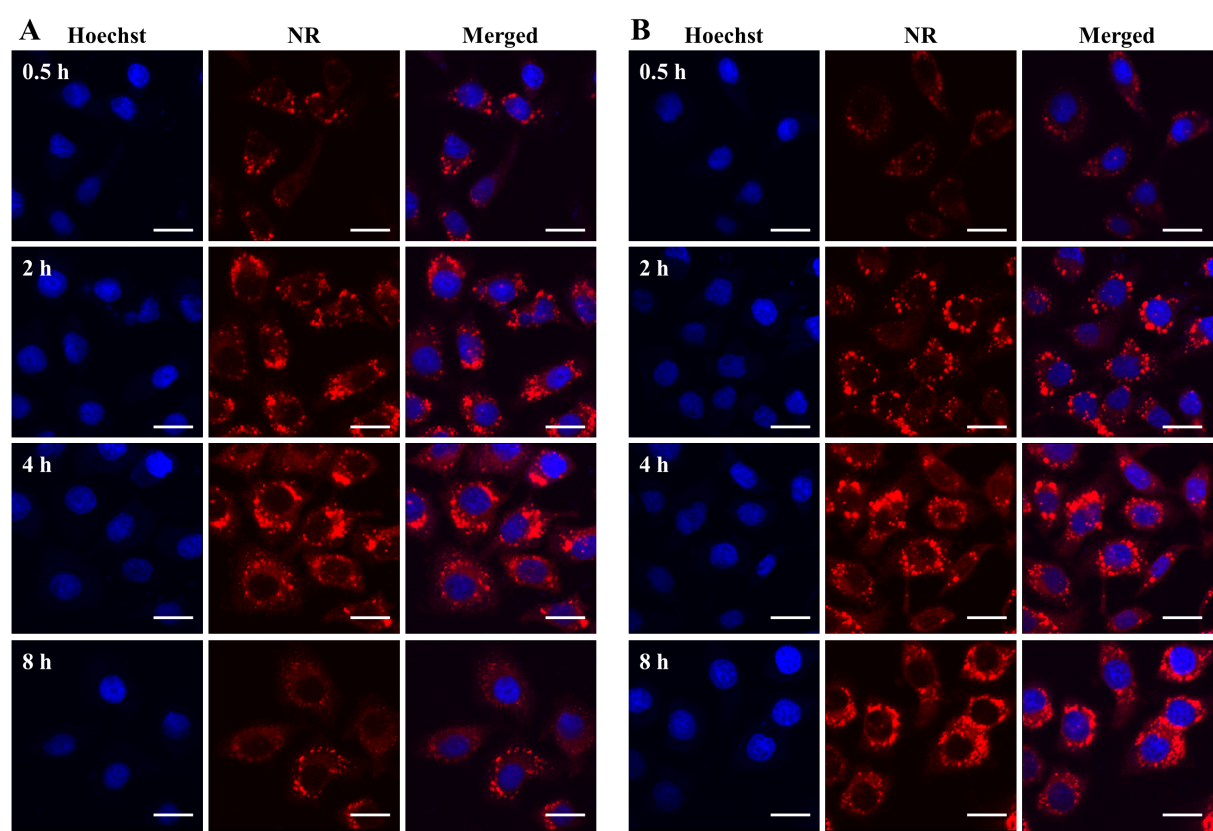


**Fig. S10.** CLSM images of HepG2 cells incubated with (**A**) SS NPs and (**B**) C6 NPs at 37 °C for different times. Scale bars, 20 *μ*m.


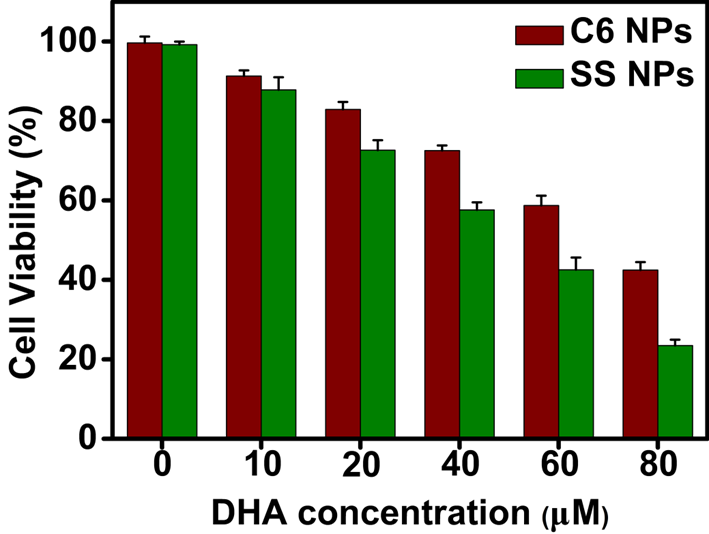


**Fig. S11.** Cell viabilities of C6 and SS NPs against HepG2 cells at different concentrations after incubation for 48 h.


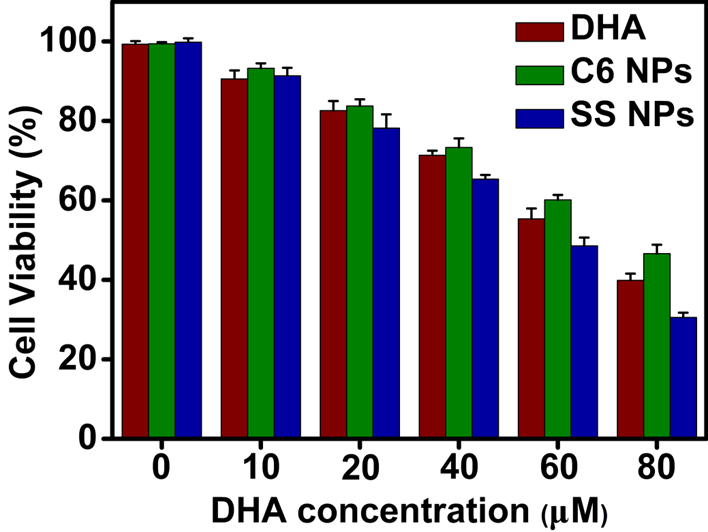


**Fig. S12.** Cell viabilities of free DHA, C6 and SS NPs against HeLa cells at different concentrations after incubation for 48 h.


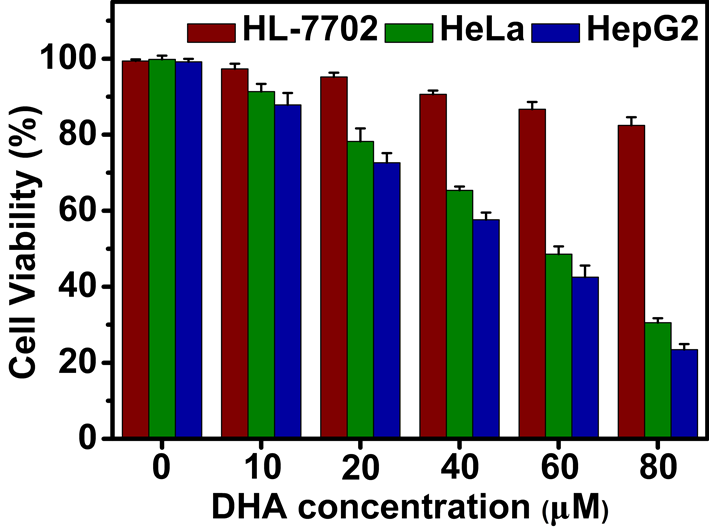


**Fig. S13.** Cell viabilities of SS NPs against HL-7702, HeLa and HepG2 cells at different concentrations after incubation for 48 h.


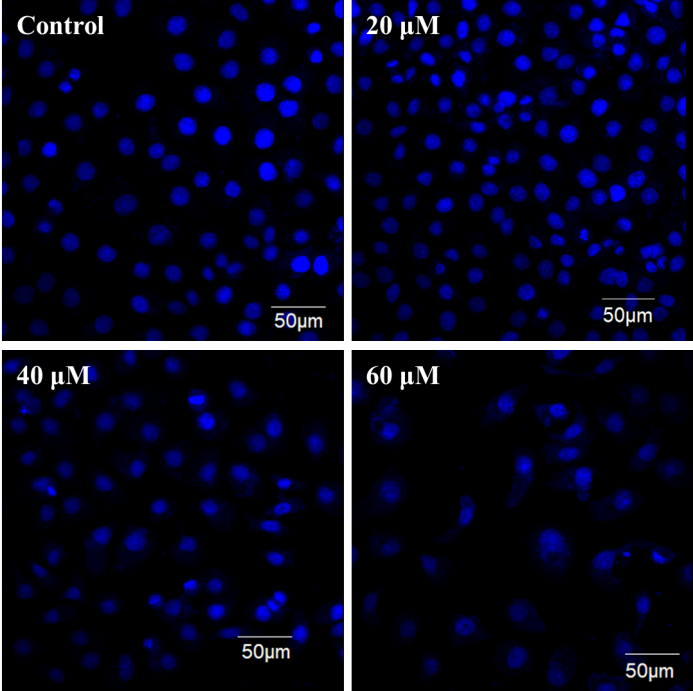


**Fig. S14.** Morphological apoptosis by staining with Hoechst 33258 in HepG2 cells treated with different concentrations of SS NPs.


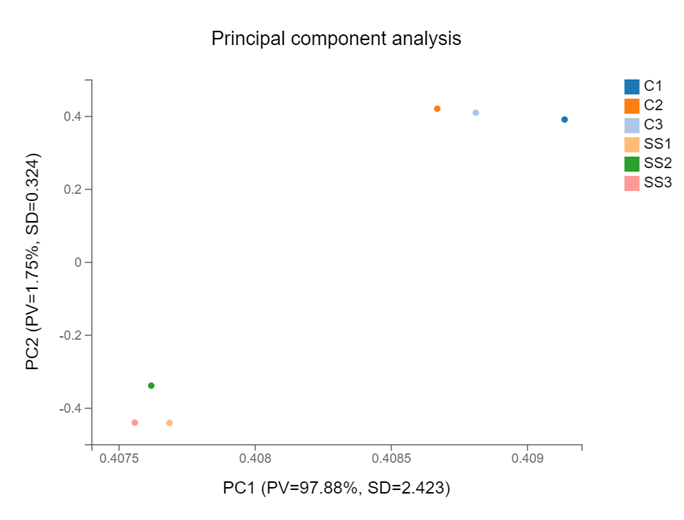


**Fig. S15.** Principal component analysis (PCA) of HepG2 cells based on untreated control group (C) and SS NPs treatment group (SS).


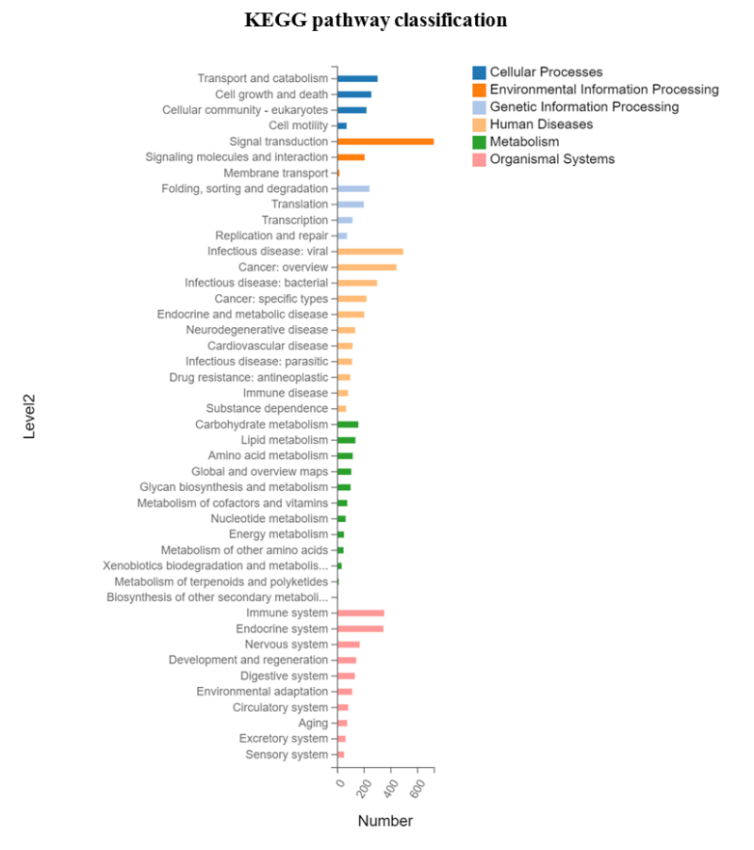


**Fig. S16**. KEGG pathway classification of differential expressed genes (DEGs). X axis represents number of DEGs, Y axis represents functional classification of KEGG.


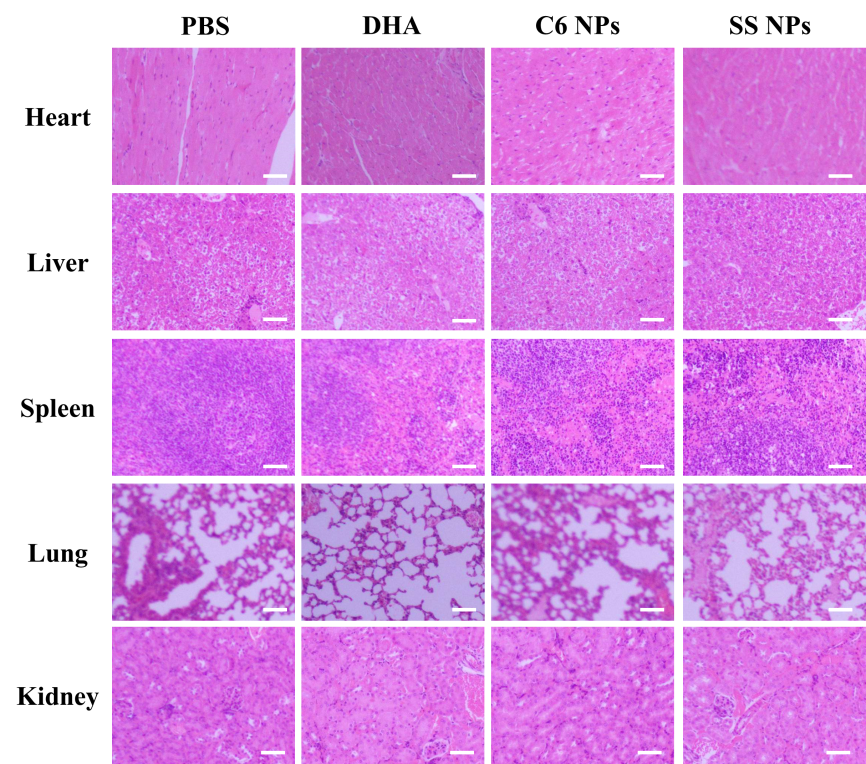


**Fig. S17.** H&E staining of the major organs (heart, liver, spleen, lung and kidney) of mice with H22 tumor xenografts after different treatments. Scale bars: 100 μm.
